# Efficacy and Safety of Adjunctive Aripiprazole LAI or Paliperidone LAI for the Management of Patients Suffering from Bipolar I Disorder with Comorbid Obsessive-Compulsive Disorder

**DOI:** 10.3390/jcm14030954

**Published:** 2025-02-02

**Authors:** Vassilis Martiadis, Enrico Pessina, Fabiola Raffone, Azzurra Martini, Matteo Di Vincenzo, Bianca Della Rocca, Domenico De Berardis, Carlo Ignazio Cattaneo, Gaia Sampogna

**Affiliations:** 1 Department of Mental Health, Asl Napoli 1 Centro, 80125 Naples, Italy; 2 Department of Mental Health, Asl Cuneo 2, 12042 Bra, Italy; 3 Department of Psychiatry, University of Campania “L. Vanvitelli”, 80138 Naples, Italy; 4 Department of Mental Health, Asl Teramo, 64100 Teramo, Italy; 5 Department of Mental Health, Asl Biella, 13900 Biella, Italy

**Keywords:** bipolar disorder, OCD, long-acting injectable antipsychotics, paliperidone, aripiprazole, pharmacological treatment, comorbidity, BD-OCD

## Abstract

**Background/Objectives**: Bipolar disorder (BD) and obsessive-compulsive disorder (OCD) often coexist, presenting significant challenges in treatment. OCD comorbidity in BD is associated with severe clinical features such as increased suicidality and impaired functioning. While selective serotonin reuptake inhibitors (SSRIs) are effective for OCD, they may increase manic shifts in BD patients. The use of long-acting injectable (LAI) antipsychotics such as monthly aripiprazole (ARI-LAI) and monthly paliperidone (PP-LAI) has been proposed as a promising alternative for BD treatment, while their efficacy and safety in BD-OCD remain understudied. This study investigated the effectiveness and tolerability of ARI-LAI and PP-LAI as adjunctive therapies in this population. **Methods**: 27 BD-OCD patients were treated with ARI-LAI or PP-LAI, alongside mood stabilizers (MS) (lithium or sodium valproate), over a 24 week period. Clinical and psychopathological assessments were performed at baseline and regular intervals using the Yale-Brown Obsessive Compulsive Scale and the Hamilton Depression Rating Scale. Safety and tolerability were evaluated using the UKU Side Effect Rating Scale. **Results**: Both treatments led to significant reductions in obsessive-compulsive symptoms and mood stabilization without manic episodes. ARI-LAI showed superior tolerability in terms of body weight gain compared to PP-LAI, with no significant differences in overall efficacy between the groups. **Conclusions**: This study is the first that investigated the efficacy and safety of adjunctive PP-LAI and ARI-LAI in this population highlighting their potential as effective and well-tolerated options for managing BD-OCD. Further studies are needed to confirm these results and refine treatment strategies for this complex population.

## 1. Introduction

Bipolar disorder (BD) is a severe mental disorder associated with high levels of personal and social disability. According to recent estimates, the incidence of BD is up to 2.4% and patients require pharmacological and psychosocial integrated and personalized management plans. In many cases, patients suffering from BD present other clinical conditions in comorbidity, including obsessive-compulsive disorder (OCD), anxiety, depressive and personality disorders. Among these comorbid conditions, OCD is quite common with a prevalence varying from 11 to 21% [1,2]. The presence of comorbid OCD in the context of BD is associated with earlier onset, increased suicidal ideation and attempts, psychotic features, rapid cycling, alcohol-related problems and impaired functioning [3,4,5]. Moreover, treating comorbid BD-OCD patients might be particularly challenging since selective serotonin reuptake inhibitors (SSRIs)–which represents the first-line treatment for OCD—may induce manic shifts, mixed states or cycle acceleration in patients with BD, especially when prescribed at high doses and for extended periods [6,7]. In particular, clomipramine and SSRIs have been associated with a 39% and 14% risk of inducing hypo/manic shifts in patients with BD [8]. More recently, a systematic review on the treatment of BD-OCD comorbidity found that pharmacological treatments for BD tend to be more effective and less harmful than OCD-specific treatments such as SSRIs [9]. Despite the scanty and heterogeneous evidence available to date, mood stabilization appears to be the primary goal in the treatment of BD-OCD patients [9].

As suggested by many international guidelines, the pharmacological management of patients with BD represents a challenge for clinicians, and several different drugs are indicated. Among the second generation antipsychotics, aripiprazole and paliperidone are commonly prescribed; both antipsychotics have demonstrated efficacy mainly in the management of manic episodes and prevention of manic swings in BD patients, while their effect on depressive polarity appears less robust [10]. According to Italian regulation, only oral prescription of atypical antipsychotics for patients with BD is considered “In label”, the use of long-acting formulation represents an off-label treatment [11].

A recent meta-analysis comparing the efficacy and safety of pharmacological treatments for acute mania showed that both oral aripiprazole and paliperidone outperformed placebo in reducing mania symptoms, with lower discontinuation rates due to ineffectiveness, and showed robust mood-stabilizing properties for the two atypical antipsychotics [12]. Moreover, aripiprazole and paliperidone are often used as augmentation strategies in OCD patients who do not respond to antidepressant treatment [13]. Some evidence suggests that second generation antipsychotics may act as mood stabilizers (MS) and provide anti-obsessive activity, which could be an effective treatment for OCD symptoms in BD patients [14]. Given that the European Medicines Agency (EMA) has not yet approved any second-generation long-acting injectable antipsychotic (LAI) for the treatment of BD, it would be interesting to investigate these pharmacological options in an Italian real-world setting. This could contribute to a deeper understanding of the clinical challenges associated with BD-OCD comorbidity.

To the best of our knowledge, there are currently no studies that have evaluated the efficacy and safety of monthly aripiprazole LAI (ARI-LAI) and monthly paliperidone LAI (PP-LAI) in comorbid BD-OCD patients. Based on these premises, the present retrospective study has been conducted in order to assess the efficacy and safety of adjunctive treatment with ARI-LAI or PP-LAI in reducing obsessive-compulsive symptomatology in a sample of real-world patients suffering from BD and comorbid OCD. The choice of LAI formulations over oral ones was based on their potential to improve treatment adherence, deliver consistent therapeutic plasma levels and reduce the risk of missed doses, which is particularly relevant in real-world settings where BD-OCD comorbidity may present additional challenges.

## 2. Materials and Methods

A retrospective design has been adopted. In particular, patients with a concurrent diagnosis of type I BD and OCD according to DSM-5 criteria [15] and fulfilling the following criteria were included: age 18–70 years, BD-OCD comorbidity, concomitant treatment with ARI-LAI or PP-LAI and sodium valproate or lithium, availability of clinical and psychopathological assessments at fixed intervals, and no concurrent antidepressant treatment. All patients were retrospectively evaluated over a 24-week period (baseline, week 4, week 8, week 12 and week 24) from prescription of ARI-LAI (300–400 mg/month) or PP-LAI (150–100 mg/month) to ongoing MS treatment (lithium or sodium valproate). ARI-LAI or PP-LAI were initiated during the acute phase of BD with concurrent moderate to severe obsessive-compulsive symptoms. Anxiolytics prescription was allowed for the short-term management of anxiety symptoms. ARI-LAI and PP-LAI dosages were consistent with those recommended for schizophrenia, as no established guidelines exist for BD-OCD patients. The dosages were chosen according to clinical judgment to achieve both mood stabilization and obsessive-compulsive symptoms reduction. Exclusion criteria were lack of clinical data before week 8 of the observation period. No other exclusion criteria were considered in order to reproduce as closely as possible the real population of BD-OCD.

Participants were recruited from the Community Mental Health Centers of Bra (northern Italy), Teramo (central Italy) and Naples (southern Italy), either by referral from a general practitioner or other psychiatrist, or by self-referral. All psychiatric diagnoses and clinical assessments, as well as decisions regarding LAI starting dosages and changes, were based on the clinical judgement of an expert psychiatrist (>10 years of clinical experience in the treatment of BD-OCD). Sodium valproate and lithium levels were monitored monthly to ensure therapeutic range (lithium: 0.5–0.8 mmol/L; sodium valproate: 50–100 mcg/L). Follow-up visits were performed according to usual clinical practice. Given the retrospective, real-world nature of the study, treatment decisions were not influenced and patients received usual outpatient care. The clinical records examined covered the period from January 2021 to July 2023.

All patients signed a written informed consent for their medical data to be treated anonymously for possible use in teaching or research. Written informed consent was also obtained for off-label treatment. The retrospective nature of the data collected did not require a precautionary request to the local Ethics Committee.

A review of medical records and interviews to patients’ referring clinicians were used to collect socio-demographic and clinical information on each subject. Control visits were conducted according to clinical practice during the 24-week follow-up period. The severity of clinical symptoms was assessed by a trained psychiatrist or psychologist using the Yale-Brown Obsessive Compulsive Scale (YBOCS) [16], the Hamilton Depression Rating Scale (HDRS) [17], the Brief Psychiatric Rating Scale (BPRS) [18], the Young Mania Rating Scale (YMRS) [19], and the Hamilton Anxiety Rating Scale (HARS) [20]. The safety and tolerability of adjunctive treatment with ARI-LAI and PP-LAI was assessed using the UKU Side Effect Rating Scale [21].

The primary outcome was the mean change in YBOCS scores from baseline to endpoint, measuring the efficacy of adjunctive ARI-LAI and PP-LAI.

General linear model for repeated measures was used to look for statistically significant changes in primary outcome during the observation period. The unpaired *t*-test was used to compare the efficacy of the two treatments. Statistical analysis was performed using the IBM SPSS^®^ software (IBM Corp. Released 2010. IBM SPSS Statistics for Windows, Version 19.0. Armonk, NY, USA). Significance was set at *p* < 0.05.

## 3. Results

The final sample consisted of 27 patients, mainly male (55.6%) with a mean age of 43.9 ± 11.5 (standard deviation, SD) years and a mean duration of the illness of 18.3 (±5.3) (Table 1). In 66.7% of cases (n = 18), patients were treated with lithium and 33.3% (n = 9) with sodium valproate; a small proportion of patients (18.5%) were prescribed anxiolytics for short-term management of anxiety symptoms with no significant inter-group difference; the bivariate analysis revealed no significant differences in socio-demographic and clinical characteristics between the two groups.

**Figure 1:**
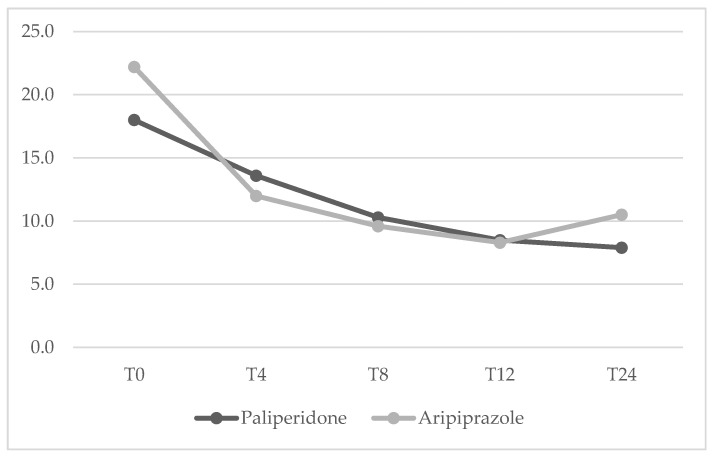
HDRS total scores in PP-LAI and ARI-LAI groups across timepoints (weeks). (GLM for repeated measures; ARI-LAI *p* < 0.01; PP-LAI *p* < 0.01; ARI-LAI vs. PP-LAI *p* > 0.5).

**Figure 2:**
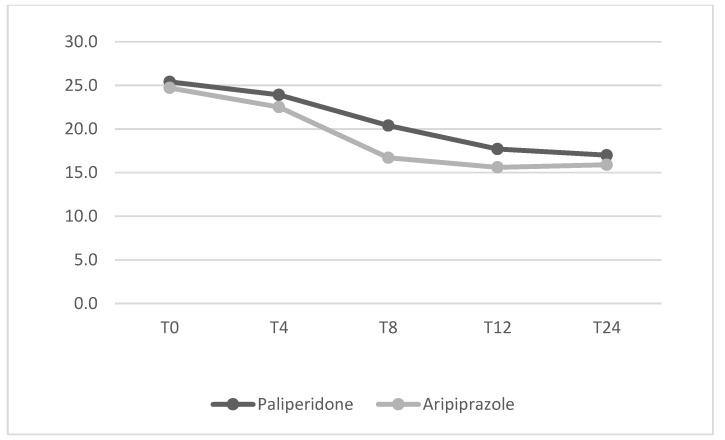
YBOCS total scores in PP-LAI and ARI-LAI groups across timepoints (weeks). (GLM for repeated measures; ARI-LAI *p* < 0.01; PP-LAI *p* < 0.01; ARI-LAI vs. PP-LAI *p* > 0.5).

**Figure 3:**
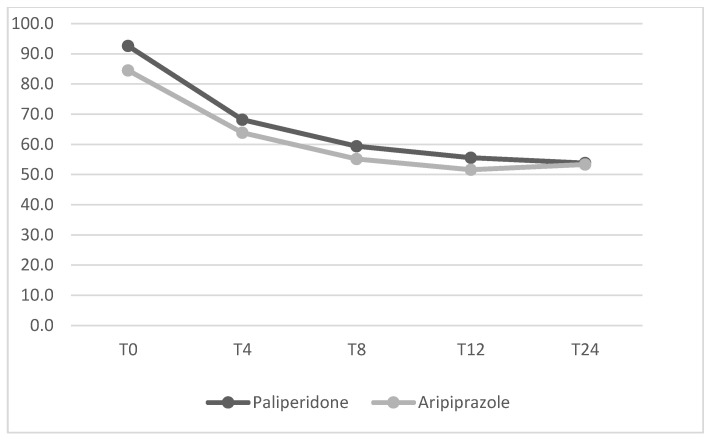
BPRS total scores in PP-LAI and ARI-LAI groups across timepoints (weeks). (GLM for repeated measures; ARI-LAI *p* < 0.001; PP-LAI *p* < 0.001; ARI-LAI vs. PP-LAI *p* > 0.5).

**Figure 4:**
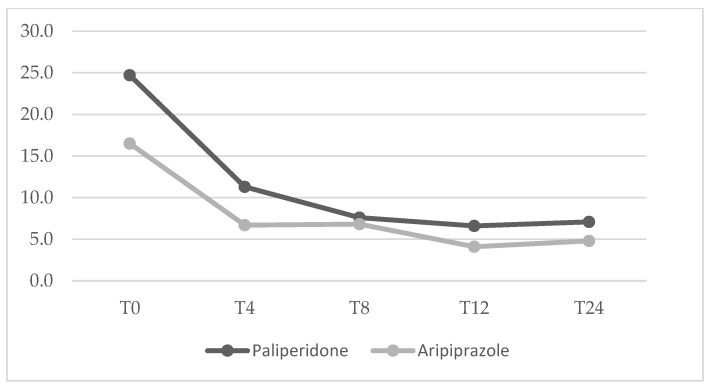
YMRS total scores in PP-LAI and ARI-LAI groups across timepoints (weeks). (GLM for repeated measures; ARI-LAI *p* < 0.01; PP-LAI *p* < 0.01; ARI-LAI vs. PP-LAI *p* > 0.5).

**Figure 5:**
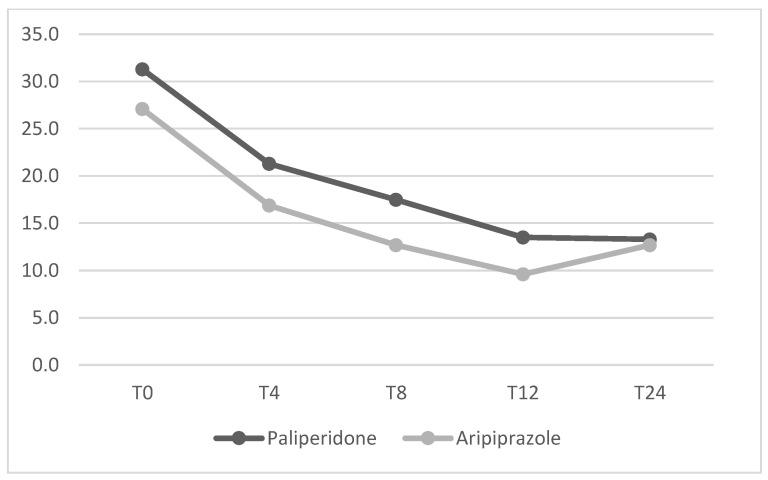
HARS total scores in PP-LAI and ARI-LAI groups across timepoints (weeks). (GLM for repeated measures; ARI-LAI *p* < 0.01; PP-LAI *p* < 0.01; ARI-LAI vs. PP-LAI *p* > 0.5).

Mean dosage of antipsychotics have been summarized in Table 2. No significant differences between the two groups were found from baseline to the 24-week endpoint for any of the psychopathological measures. The mean changes in HDRS, YBOCS, BPRS, YMRS and HARS total scores from baseline to endpoint (T24) are shown in Figure 1, Figure 2, Figure 3, Figure 4 and Figure 5 respectively. Changes in body weight (BW) and body mass index (BMI) from baseline to endpoint (T24) are summarized in Table 3. BW for the PP-LAI group increased from 77.3 ± 19.6 kg to 81.5 ± 19.7 kg, while BW for the ARI-LAI group increased from 70.1 ± 22.9 to 70.9 ± 22.3. The difference between the groups in the mean change in BW (kg) was statistically significant (4.2 ± 2.2 kg for the PP-LAI group vs. 0.8 ± 3.8 kg for the ARI-LAI group, *p* value = 0.007). BMI increased from 26.1 ± 5.4 to 27.4 ± 5.5 m^2^/kg for patients in the PP-LAI group and from 24.2 ± 7.6 to 24.6 ± 7.0 m^2^/kg for patients in the ARI-LAI group. A statistically significant difference was found in the percentage change in BMI between the two groups (5.5 ± 2.8% for the PP-LAI group vs. 1.7 ± 6.1% for the ARI-LAI group, *p* value = 0.036).

## 4. Discussion

The clinical management of patients with BD and comorbid OCD represents a relevant challenge for ordinary practice, due to the risk of pharmacological interactions among different drugs to be used. SSRIs, representing the first-line treatment for OCD—have been associated to the risk for hypo/manic switch, particularly at higher doses or over extended periods [8], and for mixed states and cycle acceleration [6,7]. Mood stabilization is widely considered to be the primary treatment goal [9]. However, evidence on the combined management of both BD and OCD remains heterogeneous. While oral atypical antipsychotics are currently used in managing BD-OCD comorbidity, there is still scope for further investigation into the potential benefits of their long-acting formulations. The safety and efficacy of second-generation LAI antipsychotics have been extensively studied in patients with schizophrenia [22,23,24]. The US Food and Drug Administration (FDA) has approved two second-generation LAI antipsychotics for the maintenance treatment of BD (risperidone microspheres, as monotherapy or add-on to a MS, and aripiprazole monohydrate, as monotherapy), whereas no second-generation LAI antipsychotic has been approved by the EMA for BD. A recent systematic review and expert consensus on the use of LAIs in patients with BD recommends the use of these formulations in people with type I BD, rapid-cycling BD and bipolar-type schizoaffective disorder. It also recommends their use in BD patients with poor adherence, multiple episodes, infrequent but severe episodes, residual symptoms while taking multiple oral medications, or a preference for LAIs over oral treatments [25]. However, despite the positive recommendations for second-generation LAI antipsychotics in BD, there are limitations and gaps in the available data on different agents. While ARI-LAIs have been tested in a large, double-blind, randomized trial with a 52-week open-label extension [26,27,28] and examined using national registry data [29], PP-LAIs have only been investigated in case series [30], a small retrospective mirror study [31] and studies using national registry data [29].

To our knowledge, this is the first study to investigate the efficacy and tolerability of PP-LAI and ARI-LAI as adjunctive therapy to MS (lithium and sodium valproate) in type I BD patients with comorbid OCD. Despite the small sample size, these results provide valuable clinical information on two long-acting antipsychotics commonly used in the treatment of BD but understudied in patients with BD and even more so in those with BD—OCD comorbidity. The majority of patients were unemployed or unable to work, which is consistent with the poor level of functioning and quality of life in patients with comorbid conditions, compared with those affected either by BD or OCD alone [32]. In line with the study by Bramante et al. [33] reporting that episodic OCD is more frequently associated with BD, in our sample the majority of the sample had an episodic OCD.

At the end of the 24-week observation period, both groups showed a significant reduction in the severity levels of obsessive-compulsive symptoms, with a good mood stability. However, a recent 12-week randomized clinical trial evaluating the safety and efficacy of oral risperidone and oral aripiprazole as adjunctive treatment for OCD in patients with BD [34] found that both oral antipsychotics can be used effectively without serious adverse effects. Another real-world prospective observational study published by Di Salvo et al. [35] reported that 12 weeks of oral aripiprazole added to lithium or sodium valproate can reduce OCD symptoms in patients with BD-OCD. In our study, the treatment response rate was 55.5% (n = 15), as measured by a ≥35% reduction in YBOCS total scores from baseline to Week 24, with no statistically significant differences between groups, demonstrating relevant efficacy for both adjunctive LAI treatments. The discontinuation LAIs have a narrow peak-to-trough fluctuation index and therefore more stable plasma levels: they are therefore thought to be better tolerated than immediate-release formulations [36]. In particular, monthly ARI-LAI have the most favorable peak-to-trough plasma concentration ratio compared to oral antipsychotics, but also to other LAIs at steady state [37]. The higher response rate may be partly explained by the higher adherence to treatment offered by LAI formulations compared with oral antipsychotics. However, this may have been influenced by sample size. Non-adherence to antipsychotics has always been a relevant issue, with an average non-adherence rate of 42% in BD patients [38]. A recent nationwide cohort study on adherence to MS and antipsychotics found a higher discontinuation rate with oral paliperidone compared to oral aripiprazole [39,40,41,42]. Our study did not find statistically significant differences between the two long-acting efficacy profiles, probably due to the limited sample size.

In terms of tolerability, there were no safety issues with treatment with either LAIs, although 48.2% of the total sample had medical comorbidity before starting antipsychotic treatment, mainly hypertension and diabetes. A significant difference in mean body weight and BMI variation was found, in contrast with Shymko et al. [43], highlighting a time-dependent increase in weight after 12 months of treatment in patients with first-episode psychosis. This difference should be due to our short follow-up period (24 vs. 52 weeks) and to the different clinical population considered. However, the impact of LAIs on BW and BMI in patients with BD deserves further studies, since weight gain represents one of the most common reasons for switching antipsychotic therapy. Our results provide clinicians with useful information about the effects of two commonly used LAIs on weight gain in a BD-OCD population that may be prone to adherence problems [44]. Regarding non-weight-related side effects, 22.2% of the total sample reported no side effects (9.1% in the PP-LAI group vs. 31.2% in the ARI-LAI group), supporting an overall good tolerability of both LAIs. While our findings suggest that both treatments were effective and well-tolerated, future studies should explore whether BD-OCD patients require different dosing strategies compared to those used in schizophrenia.

The present study has some limitations, which should be acknowledged. First, the small sample size may have influenced our results and limited their generalizability. Second, the limited follow-up period and the retrospective data collection should have coupled with data collected in a longitudinal real-world study. Third, the lack of routine laboratory tests in the ordinary clinical practice prevented the systematic evaluation of endocrine side effects. Future studies should include prolactin measurements and evaluation of metabolic tolerability to provide a more comprehensive safety profile of ARI-LAI and PP-LAI in BD-OCD patients.

## 5. Conclusions

Patients with BD and comorbid OCD present a unique clinical challenge due to the complex interplay between these conditions and the risks associated with pharmacological treatments. This study is the first to investigate the efficacy and safety of adjunctive ARI-LAI and PP-LAI, in this population. The results demonstrate significant reductions in obsessive-compulsive symptoms and robust mood stabilization over a 24-week period, without evidence of hypomanic or manic shifts, underscoring the potential of LAI antipsychotics as effective and well-tolerated treatment options for BD-OCD. Key strengths of this study include its focus on a challenging and understudied population, the real-world setting providing practical insights for clinicians, and the detailed assessment of both efficacy and safety outcomes. Additionally, the findings provide valuable information on weight-related tolerability, which is a crucial factor influencing adherence in bipolar patients. The present study should be useful for promoting further rigorous, longitudinal and multicentric studies on the efficacy and effectiveness of LAI antipsychotics in treating patients with BD-OCD.

## Figures and Tables

**Table 1:** Participants’ socio-demographic and clinical characteristics.

	Global Sample (n = 27)	PP-LAI (n = 16)	ARI-LAI (n = 11)
Gender, female, % (N)	44.4 (12)	37.5 (6)	54.5 (6)
Age, M (SD)	43.9 (11.5)	44.6 (11.7)	42.8 (11.7)
Living situation, % (N) Single Married Separated Widowed	70.4 (19) 11.1 (3) 14.8 (4) 3.7 (1)	81.3 (13) 6.3 (1) 12.5 (2) 0 (0)	54.5 (6) 18.2 (2) 18.2 (2) 9.1 (1)
Children, yes, % (N)	25.9 (7)	18.8 (3)	36.4 (4)
Years of education, M (SD)	13.0 (1.2)	11.9 (0.7)	14.7 (2.7)
Occupation, % (N) Unemployed Blue collar Homemaker Farmer Clerk Student Retired Unable to work	22.2 (6) 22.2 (6) 3.7 (1) 3.7 (1) 3.7 (1) 3.7 (1) 7.4 (2) 33.3 (9)	31.3 (5) 12.5 (2) 6.3 (1) 6.3 (1) 6.3 (1) 0 (0) 6.3 (1) 31.3 (5)	9.1 (1) 36.4 (4) 0 (0) 0 (0) 0 (0) 9.1 (1) 9.1 (1) 36.4 (4)
Suicide attempt, yes, % (N)	48.1 (13)	43.8 (7)	54.5 (6)
Psychiatric comorbidities, % (N) No Anxiety Gambling Anorexia nervosa Dysmorphophobia Skin picking	63.0 (17) 3.7 (1) 7.4 (2) 11.1 (3) 11.1 (3) 3.7 (1)	62.5 (10) 0 (0) 12.5 (2) 12.5 (2) 12.5 (2) 0 (0)	63.6 (7) 9.1 (1) 0 (0) 9.1 (1) 9.1 (1) 9.1 (1)
Mood stabilizer % (N) Lithium Valproate	66.7 (18) 33.3 (9)	62.5 (10) 37.5 (6)	72.7 (8) 27.3 (3)
OCD Course % (N) Episodic Chronic	62.9 (17) 27.1 (10)	56.2 (9) 33.8 (7)	81.8 (9) 18.2 (2)

**Table 2:** LAI antipsychotics dosages across timepoints (weeks).

	T0 (n = 27)	T4 (n = 27)	T8 (n = 26)	T12 (n = 24)	T24 (n = 22)
PP-LAI, yes, % (N) 50 mg 75 mg 100 mg 150 mg	0 (0) 0 (0) 62.5 (10) 37.5 (6)	0 (0) 25 (4) 50 (8) 25 (4)	20.0 (3) 13.3 (2) 53.3 (8) 13.3 (2)	23.1 (3) 7.7 (1) 53.8 (7) 15.4 (2)	25.0 (3) 8.3 (1) 50.0 (6) 16.7 (2)
ARI-LAI yes, % (N) 300 mg 400 mg	0 (0) 100 (11)	0 (0) 100 (11)	9.1 (1) 90.9 (10)	9.1 (1) 90.9 (10)	10.0 (1) 90.0 (10)

**Table 3:** Body weight and BMI variations in PP-LAI and ARI-LAI groups.

	PP-LAI (n = 16) Mean (DS)	ARI-LAI (n = 11) Mean (DS)	*p* Value
BW T0 (kg)	77.3 (19.6)	70.1 (22.9)	0.388
BW T24 (kg)	81.5 (19.7)	70.9 (22.3)	0.206
BMI T0	26.1 (5.4)	24.2 (7.6)	0.446
BMI T24	27.4 (5.5)	24.6 (7.0)	0.242
Mean BW variation (kg)	4.2 (2.2)	0.8 (3.8)	0.007
Mean BW variation (%)	5.6 (2.7)	1.6 (6.2)	0.029
BMI variation (m^2^/kg)	1.3 (0.9)	0.4 (1.7)	0.073
BMI variation (%)	5.5 (2.8)	1.7 (6.1)	0.036

## Data Availability

Data are available on reasonable request.

## Abbreviations

The following abbreviations are used in this manuscript:

BD	Bipolar disorder
OCD	Obsessive-compulsive disorder
SSRI	Selective serotonin reuptake inhibitor
LAI	Long-acting injectable antipsychotic
ARI	Aripiprazole
PP	Paliperidone
YBOCS	Yale-Brown Obsessive Compulsive Scale
HDRS	Hamilton Depression Rating Scale
BPRS	Brief Psychiatric Rating Scale
YMRS	Young Mania Rating Scale
HARS	Hamilton Anxiety Rating Scale
BW	Body weight
BMI	Body mass index
SD	Standard deviation
FDA	Food & Drug Administration
EMA	European Medicines Agency
